# Learned helplessness paradigm in P2xr7 wild type and knock out mice and its effect on synapses in the dentate gyrus

**DOI:** 10.1186/2193-1801-4-S1-P33

**Published:** 2015-06-12

**Authors:** Lilla Otrokocsi, Flóra Gölöncsér, Beáta Sperlágh, Ágnes Kittel

**Affiliations:** Institute of Experimental Medicine, Hungary

**Keywords:** p2x7, depression, spine synapse

Although depression is one of the prevailing central nervous system disorders, there is still not enough knowledge about the exact alterations underlying its pathophysiology and the treatment is often inefficient. Our study aims at exploring a potential relationship between major depressive disorder and the purinergic P2X7 receptor (P2X7R), since in previous behavioural tests P2rx7 knock out mice displayed an antidepressant-like phenotype. Among many other known symptoms, the loss of hippocampal spine synapses is a revealing feature of the disorder. Therefore we wanted to measure the density of spine synapses in the molecular layer of the dentate gyrus in the learned helplessness paradigm and later see if genetic inhibition of P2X7R could have influence on this condition. Wild type C57Bl/6 and P2rx7 knock out male mice were exposed to inescapable footshocks (IES) in shuttle boxes during 2 training days and on the 3rd day learned helplessness was tested, where helpless animals usually failed to escape. Control animals were also placed in shuttle boxes but did not receive footshocks until testing. Escape failures and the latency to escape were measured to determine helpless behaviour. Electron microscopy analysis was performed to determine spine synapse density in the different groups. In wild type mice both average escape latency and the number of failed escapes were significantly higher in the IES treated group, however, we could not find such divergency in P2rx7 knock out mice. Electron microscopy analysis confirmed alterations in spine synapse density in the molecular layer of the dentate gyrus subsequent to learned helplessness experiments, as results indicated a significant decrease in spine synapse density in wild type mice, but not in knock out animals. These findings may lead to presume a role of the P2X7 receptor in this disorder and further experiments will hopefully help get a better understanding and thus more effective treatment of major depression.

